# Comparing functional annotation analyses with Catmap

**DOI:** 10.1186/1471-2105-5-193

**Published:** 2004-12-09

**Authors:** Thomas Breslin, Patrik Edén, Morten Krogh

**Affiliations:** 1 Complex Systems Division, Department of Theoretical Physics, Lund University, Lund, Sweden

## Abstract

**Background:**

Ranked gene lists from microarray experiments are usually analysed by assigning significance to predefined gene categories, *e.g*., based on functional annotations. Tools performing such analyses are often restricted to a category score based on a cutoff in the ranked list and a significance calculation based on random gene permutations as null hypothesis.

**Results:**

We analysed three publicly available data sets, in each of which samples were divided in two classes and genes ranked according to their correlation to class labels. We developed a program, Catmap (available for download at ), to compare different scores and null hypotheses in gene category analysis, using Gene Ontology annotations for category definition. When a cutoff-based score was used, results depended strongly on the choice of cutoff, introducing an arbitrariness in the analysis. Comparing results using random gene permutations and random sample permutations, respectively, we found that the assigned significance of a category depended strongly on the choice of null hypothesis. Compared to sample label permutations, gene permutations gave much smaller *p*-values for large categories with many coexpressed genes.

**Conclusions:**

In gene category analyses of ranked gene lists, a cutoff independent score is preferable. The choice of null hypothesis is very important; random gene permutations does not work well as an approximation to sample label permutations.

## Background

In genome-wide microarray experiments, it is possible to analyse the relevance of many different categories of genes, obtained from prior knowledge in the form of database annotations or from other experiments. These gene annotation analyses can unravel new information about pathways and cellular functions responsible for different phenotypes. Computational tools aiding in this process have recently been developed [1-8], most notably for annotations based on the Gene Ontology (GO) [9]. Generally, category relevance is calculated as the *p*-value of a score, thus being dependent on both the choice of score and the choice of null hypothesis.

In microarray analyses such as clustering, which provide defined subsets of genes with no internal ranking, it is natural to base the score on the number of category genes in the relevant subset. However, ranking of genes appear in many techniques for microarray analysis, such as correlation of gene expression to target profiles [10] and scoring of genes by their ability to discriminate between experimental conditions [11-13]. A separation of relevant and irrelevant genes can easily be constructed from ranked gene lists by introducing a cutoff, but the choice of cutoff becomes somewhat arbitrary and information in the list is lost. Tools addressing this problem, by using rank-based scores that are independent of a rank cutoff, have adopted the Kolmogorov-Smirnov score [14-17], and a minimized cutoff-based *p*-value [7,8], which optimizes the cutoff for each category. The Wilcoxon rank sum [18], investigated here, serves the same purpose.

To calculate a *p*-value for the assigned score, a set of gene lists, ranked according to a chosen null hypothesis, are needed. The simplest choice of null hypothesis is just random gene permutations, and for some rank-based scores, the *p*-value can then be calculated analytically, without explicitly performing the permutations. However, the random gene permutations null hypothesis assumes independence of gene expression over biological samples, and the *p*-value is thus a combination of the *p*-value of how important the category is and the *p*-value for the genes of the category being coexpressed. When category genes behave similarly over a wide range of experimental conditions, the coexpression does not indicate relevance of the category for the question under study. In many analyses, a more appropriate null hypothesis is therefore sample label permutations, in which a set of ranked gene lists are generated based on the gene expression correlations to randomly permuted target values of the samples. This approach accounts for correlations between category genes and gives *p*-values that are bounded from below by the number of possible permutations of the samples in the data set. The latter is particularly important in data sets with few samples. Despite this, publicly available tools for gene annotation analysis are restricted to gene permutations [1-8].

We present a program, Catmap, for gene category analysis based on ranked gene lists. The program uses either the number of genes above a cutoff or the Wilcoxon rank sum as score, and the significance of the score can be calculated from a user supplied set of ranked lists, thus allowing for sample label permutations. Furthermore, the program calculates corrections for multiple category testing, using permutation results to assess an effective number of independent categories, which enables Catmap to estimate very small multiple category *p*-values, that would otherwise have been computationally infeasible. The input to the program is two files and some arguments. The first file contains the biologically relevant ranked list of genes and, if needed, additional ranked gene lists drawn from the null hypothesis. The second file contains the categories and their corresponding genes. The input arguments can either be specified on the command line or in a settings file, and are as follows: 1) a choice between the cutoff score the Wilcoxon rank sum score; 2) a choice of null hypothesis, which can be either the above mentioned user-supplied ranked lists or random gene permutations; 3) the number of permutations used in multiple category testing. If zero, no multiple category testing is performed.

The output of Catmap is two files. The main output file contains all the categories, one on each line ordered according to their significance. The line of a category contains the *p*-value, the multiple comparison *p*-value, the false discovery rate, the ROC area (which is a normalized way to represent the Wilcoxon rank sum), the number of genes in the category, and the 25th, 50th, and 75th percentiles of the ranks. The other output file, the companion file, contains all the categories, with all the genes and their ranks listed below. Each line contains a gene and its rank. The program can be downloaded at [19], where file format specification and example files are accessible as well.

## Results and discussion

### Comparing cutoff independent and cutoff-based score functions

We analysed the breast cancer data set of van 't Veer *et al*. [13] with a cutoff-based score function, using different cutoffs. Table 1 presents results for 15 categories with low *p*-values from cutoff independent scoring, showing that the *p*-value depends strongly on the choice of cutoff. This is further illustrated by the very different cutoffs at which the minimized cutoff-based *p*-value was obtained. A table with all categories is provided as a supplement [see Additional file 2].

Compared to the variations between the cutoff-based alternatives, the results shown in Table 1 are in reasonable agreement for two cutoff independent *p*-values, using the Wilcoxon rank sum and the minimized cutoff-based *p*-value, respectively. The *p*-value based on the Wilcoxon rank sum was most often larger than the minimal cutoff-based *p*-value. Since the latter is biased by a minimization process, it must be interpreted as a score, rather than a *p*-value, thus requiring additional analyses to find statistical significance [7,8].

### Comparing null hypotheses

**Figure 1:**
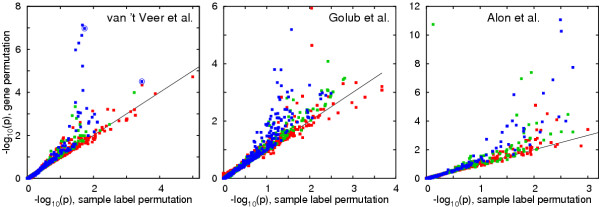
**Comparing null hypotheses**. Comparison of *p*-values obtained by sample label permutations and gene permutations, using the data set of van 't Veer *et al*. [13] (left), Golub *et al*. [11] (middle), and Alon *et al*. [20] (right). Sample label permutation results were obtained with 100.000 permutations for the van 't Veer *et al*. data set and with 10.000 permutations for the other data sets. Gene permutation results were calculated as described in Methods. Red, green and blue colours represent categories with 1 to 5, 6 to 20, and over 20 genes, respectively. Encircled boxes in the left figure represent the categories "M phase" and "carboxylic acid metabolism", which are further discussed in the text.

**Figure 2:**
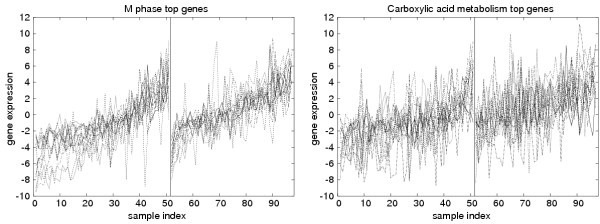
**Effects of coexpression on different null hypotheses**. Expression profiles, over the 97 samples in van 't Veer *et al*. [13], of the 12 most highly ranked genes in the "M phase" category (left) and 13 most highly ranked genes in the "carboxylic acid metabolism" category (right), respectively. Some genes were inverted since the ranking was based on absolute correlation values to metastasis class. The metastasis free samples are to the left of the vertical line, and within each metastasis class, samples are ordered in increasing average expression of the examined genes. The expressions of each gene was normalized to zero average across samples. The narrower band of expressions in the left figure illustrates the higher Pearson correlation of M phase genes. Average absolute Pearson correlation between gene expressions was 0.74, with standard deviation of 0.16, for the M phase genes, and 0.44, with standard deviation of 0.27, for carboxylic acid metabolism genes.

Using the Wilcoxon rank sum, we compared the results of different null hypotheses. Three publicly available data sets were examined [11,13,20]. As can be seen in Figure 1, *p*-values based on gene permutations tend to be lower than those based on sample label permutations. For categories with small *p*-values, there are remarkable differences, in particular for large categories with more than 20 genes. Since the gene permutation null hypothesis assumes independent genes, we expect a GO category whose genes are uncorrelated to have roughly the same *p*-value under the two different null hypotheses, whereas a significant category whose genes are highly correlated will get a lower *p*-value using the gene permutation null hypothesis. To illustrate this coexpression effect, we picked two large categories, "carboxylic acid metabolism" and "M phase", which are encircled in Figure 1. In the data set of van 't Veer *et al*. [13], "carboxylic acid metabolism" has similar *p*-values for the two null hypotheses, while "M phase" has a *p*-value of 10^-7^using gene permutations but the much higher *p*-value of 3 · 10^-2 ^using sample label permutations. As seen in Figure 2, the most highly ranked genes of "M phase" are indeed more coexpressed than the most highly ranked genes of "carboxylic acid metabolism".

In Table 2, the ranks of categories for the different null hypotheses are compared. There are distinct differences, with only a small overlap among top ten categories. One can clearly see the tendency for the gene permutation null hypothesis to find categories with very many genes, as discussed above. A table with all categories is provided in the supplement [see Additional file 3].

Table 2 also shows category ranks obtained with two alternative cutoff independent score functions: the Kolmogorov-Smirnov score as used in GSEA [17] and the minimal cutoff-based *p*-value used in FuncAssociate [7] and iGA [8]. These two alternatives do not calculate *individual p*-values for categories, but ranks categories based on the chosen score. Nevertheless, they give results similar to those obtained with the Wilcoxon rank sum and gene permutation. This is expected, since the minimized *p*-value is calculated with gene permutations, and the score adopted in GSEA [17] ranks categories similarly to what a Kolmogorov-Smirnov *p*-value, based on gene permutations, would do. It should be noted that GSEA, FuncAssociate, and iGA calculate multiple hypotheses corrected *p*-values, but these do not change the ranking of categories.

There is a possible difference (which does not reveal itself in Table 2) between the Kolmogorov-Smirnov score and minimized *p*-value score on one hand, and the Wilcoxon rank sum on the other, in the treatment of categories for which only a subset of genes have expressions correlating significantly with the question under study. The important genes being in the top of the ranked list will give the category a good score with all three score functions, provided the remaining, seemingly insignificant, genes are distributed in the ranked list as expected by random. However, if these less important genes lie higher in the list than expected by random (though not high enough to affect the Kolmogorov-Smirnov or min-*p *scores), the category will be considered more important by the Wilcoxon rank sum. Reversely, if the less important category genes prevail in the bottom of the list, the Wilcoxon rank sum score function will deem the category as unimportant, while the other two scores will give the category a high significance, based on the top ranked genes alone. Whether seemingly insignificant genes being ranked better or poorer than explainable by random expectations should be observed or ignored is of course a matter of taste, and a possibility is to use several score functions, that may complement each other. The differences are, however, much smaller than those related to choice of null hypothesis, as revealed in Table 2.

### Multiple category testing

The more categories that are being tested, the more likely it is that at least one category gets a very small *p*-value by chance. To better evaluate the statistical significance of the best scoring categories, we used Catmap to calculate false discovery rates and family-wise error rates by permutation tests. This also gave us an effective number of independent categories, *N*_eff_, as described in Methods.

The GO contains many small categories which would be reasonable to ignore in a study aiming at biological conclusions, and they were included in Figure 1 mainly to highlight the differences between the null hypotheses. When performing multiple category testing, we restricted the study to large categories, containing more than 20 genes. We tested the 3 sub-ontologies (biological process, molecular function, and cellular component) both separately and together.

As expected from the discussion above, several categories with coexpressed genes got small *p*_multiple _and small false discovery rates with random gene permutations. In contrast, when using sample label permutations, the smallest *p*_multiple _was obtained in the data set of van 't Veer *et al*. [13] for the biological process category "organic acid metabolism", which contained 83 genes and had p = 3 · 10^-4 ^and *p*_multiple _= 0.02. Interestingly, organic acid metabolism is known in the literature to be relevant for breast cancer [21,22]. For this data set and the biological process categories, there was a 38% false discovery rate among the top 15 categories.

For all 3 sub-ontologies, the effective number of categories, *N*_eff_, was around half of the full number of categories, *N*. In the data set of van 't Veer *et al*. [13] the numbers were *N*_eff _= 83 versus *N *= 166 for biological process, *N*_eff _= 69 versus *N *= 119 for molecular function, and *N*_eff _= 22 versus *N *= 42 for cellular component. For all categories together the real number of large categories was *N *= 327 whereas *N*_eff _= 152. Using random gene permutations for the same data set and categories, we got *N*_eff _= 170. The fact that *N*_eff _for the two null hypotheses are so close is a general phenomena that we see in all our examples (data not shown). Furthermore, for all data sets and ontologies studied, *N*_eff _was approximately half of the total number of categories. If this is a general feature for GO categories, the simple Bonferroni correction would not be totally unreasonable for small *p*-values.

**Figure 3:**
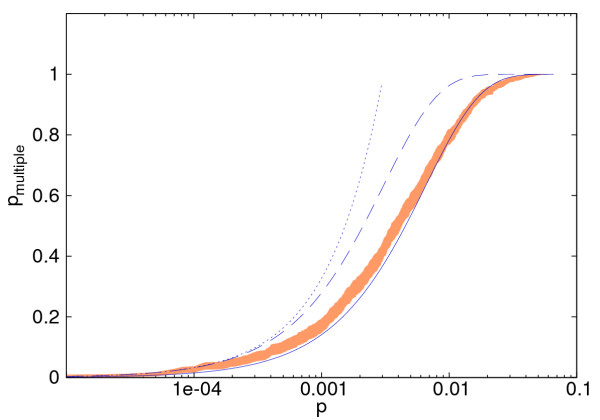
**Fitting an effective number of independent categories**. The multiple category *p*-value, *p*_multiple_, versus *p*-value for the data set of van 't Veer *et al*. [13], using 327 large Gene Ontology categories with more than 20 genes. The yellow band shows 95% confidence interval of sample label permutation results, based on 1000 random lists, and the blue curves show the results of Equation (1), with the fitted *N*_eff _= 152 (solid line), the total number of categories *N *= 327 (dashed line), and also the Bonferroni correction (dotted line).

Figure 3 shows that the fit with an effective number of categories was good; in the range where permutations results were available it did not deviate more than a factor of two. The example in Figure 3 was obtained with 100.000 sample label permutations, and minimal *p*-values were found for 1000 random gene lists.

It should be noted that whenever several ranked lists are examined as part of a project, this additional source of multiple hypotheses testing should also be corrected for. An example of such a correction, for cutoff-based score functions, is presented by Corà *et al*. [23].

## Conclusions

We developed a computer program for calculating the significance of gene categories in a ranked list of genes. Corrections for multiple category testing can be performed by the program. To investigate the properties of different scores and null hypotheses, we analyzed three publicly available data sets [11,13,20]. Commonly [1-6], a subset of relevant genes is defined from a ranked gene list by introducing a cutoff in the list. Our results show that the obtained *p*-values of biologically relevant categories depend strongly on the choice of cutoff. The cutoff independent Wilcoxon rank sum score overcomes the problem, representing an alternative to the Kolmogorov-Smirnov score [14-17] and the minimized cutoff-based *p*-value [7,8]. The ranking of categories for the three cutoff independent scores are very similar.

Though sample label permutations in many situations represent a better null hypothesis than gene permutations, available gene annotation analysis tools are restricted to the latter. Our implementation allows for both null hypotheses, and we find that both the *p*-values and the ranking of categories depend strongly on the choice of null hypothesis. Compared to sample label permutations, gene permutations gave much smaller *p*-values for large categories with many coexpressed genes.

## Methods

### Algorithm

The implemented algorithm treats the categories sequentially and independently. As score function for category relevance, the program uses either the Wilcoxon rank sum or the number of genes above a given cutoff in the ranked list. The latter is implemented for method comparison and for the case of a defined subset of relevant genes, without internal ranking.

For the case of the Wilcoxon rank sum, the user can supply a set of ranked lists distributed according to an appropriate null hypothesis, or request random permutations of genes as the null hypothesis. In the latter case, the significance of the score is calculated analytically by the program, using either an exact calculation by an iterative method, a Gaussian approximation, or a continuous volume approximation. The program chooses method based on a balance between accuracy and computation time. Details are presented in supplementary information [see Additional file 1].

For the case of the cutoff-based score function, the *p*-value of category relevance is determined with Fisher's exact test [24], corresponding to randomly permuted genes as null hypothesis.

When *N *independent categories are tested simultaneously, family-wise error rate simply means calculating the probability,

*p*_multiple _(*q*) = 1 - (1 - *q*)^*N*^,     (1)

that at least one category has a *p*-value below any given number *q *by chance. For correlated categories, we make the assumption that the same functional form describes *p*_multiple _(*q*), with *N *replaced by an effective number of independent categories *N*_eff_. We find *N*_eff _by generating a number, *K*, of ordered lists under the null hypothesis and calculating the *p*-values of all categories. We fit *N*_eff _using the maximum likelihood estimation


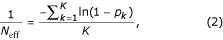


where *p*_*k *_is the minimal *p*-value for the *k*'th ordered list.

The false discovery rate for the *j *highest ranked categories is found by counting the number of *p*-values from *K *permuted lists lower than the *p*-value of the *j*:th category and divide this number with *K *· *j*. For the case of sample label permutations, when a user supplied set of ranked gene lists are used to represent the null hypothesis, the first *K *lists are used to find *N*_eff _and false discovery rates, and the remaining lists are used to calculate *p*-values for each of the *K *lists.

### Implementation

The algorithm is implemented in the Perl program Catmap.pl and is released under the GNU General Public License (GPL). Catmap.pl, together with user instructions, is available for download at [19].

### Public data sets

Using Catmap, we analysed three publicly available data sets with gene annotations from the Gene Ontology.

The data set of van 't Veer *et al*. [13] consists of 97 patients with primary sporadic breast cancer, of which 46 had metastases within five years following treatment. Quality filtering was performed as described in [13], and rendered about 5,000 genes which were ranked according to their absolute Pearson correlation to metastasis class. A Gene Ontology analysis of the data set has previously been performed with the 231 top genes as the subset of important genes and random gene permutations [25].

The data set of Golub *et al*. [11] consists of bone marrow samples from leukemia patients, 27 with AML and 11 with ALL. The published data contains expression levels for 5000 genes, which after removal of genes with no variance across samples rendered 4812 genes which were ranked according to their absolute Pearson correlation to leukemia type.

The data set of Alon *et al*. [20] consists of 40 tumour and 22 normal colon tissue samples. The 2000 genes in the published data set were ranked according to their absolute Pearson correlation to tissue type.

### Gene ontology associations

All genes were first mapped to corresponding UniGene clusters [26]. For the data set of Golub *et al*. [11] this mapping was given from chip annotation files provided by Affymetrix, whereas for the other data sets [13,20], the mapping was done via GenBank accession numbers. GO annotations for UniGene clusters were extracted with ACID [27], and completed by back propagating all lower level associations on the GO graph.

## Authors' contributions

TB and MK implemented the algorithms in Catmap. All authors participated in the design of the study, prepared, read, and approved the final manuscript.

## Supplementary Material

Additional File 2 supplement to Table 1. Click here for file

Additional File 3 supplement to Table 2. Click here for file

Additional File 1 *p*-values for the Wilcoxon rank sum score. Click here for file

## Figures and Tables

**Table 1:** Category *p*-values for cutoff-based and cutoff independent score functions. The 15 GO categories with the lowest Wilcoxon rank sum *p*-values from the ranked gene list, based on the data set of van't Veer *et al*., which comprises 5224 genes in total. Three columns show *p*-values for cutoff based score functions, with cutoffs at position 100, 300 and 600 in the list. The columns "min *p*" and "cutoff" give the minimal cutoff based *p*-value and the cutoff where this minimum was attained. The column "WRS" gives the *p*-value calculated with the Wilcoxon rank sum as score function and random permutation of genes as null hypothesis, and the column marked #genes indicates the total number of genes in the category. A full table (sorted by WRS *p*-value) is given as supplementary information [see Additional file 2]. The supplementary table also contains the ranking of each category using the different methods and the 25th, 50th and 75th percentiles of those genes in the ranked list.

GoName	GoId	top100	top300	top600	min *p*	cutoff	WRS *p*	#genes
mitotic cell cycle	0000278	9e-05	5e-10	6e-07	3e-10	290	7e-08	93
M phase	0000279	1e-03	2e-06	1e-04	1e-09	1491	1e-07	41
nuclear division	0000280	1e-03	2e-06	1e-04	4e-09	1491	2e-07	40
M phase of mitotic cell cyc..	0000087	6e-04	5e-07	1e-04	2e-08	1491	5e-07	36
mitosis	0007067	5e-04	4e-07	1e-04	6e-08	1491	1e-06	35
cell cycle	0007049	2e-04	3e-07	1e-05	1e-07	1571	6e-06	172
carbon-nitrogen ligase act..	0016884	4e-04	3e-03	1e-02	3e-05	27	2e-05	2
carboxylic acid metabolism	0019752	9e-02	1e-04	4e-05	3e-06	711	3e-05	83
organic acid metabolism	0006082	9e-02	1e-04	4e-05	3e-06	711	3e-05	83
intramolecular isomerase ..	0016863	1e+00	3e-02	8e-04	2e-05	609	5e-05	4
cell proliferation	0008283	7e-04	2e-05	1e-03	4e-06	1956	8e-05	264
intramolecular isomerase ..	0016860	1e+00	2e-02	3e-03	3e-05	905	1e-04	9
spindle microtubule	0005876	4e-04	3e-03	1e-02	6e-05	42	1e-04	2
DNA replication and chro..	0000067	6e-02	3e-04	2e-03	9e-05	852	3e-04	44
regulation of mitosis	0007088	9e-03	8e-03	5e-02	3e-04	1248	5e-04	8

**Table 2:** Comparison of different cutoff independent approaches. The top ten categories and their corresponding ranks for each of the the four methods: Wilcoxon rank sum (WRS) with sample label permutation null hypothesis (s.l.p.), WRS with gene permutation null hypothesis (g.p.), the Kolmogorov-Smirnov score (K-S) as used in GSEA [17], and the minimal cutoff-based *p*-value (min-*p*) [7, 8]. Percentile columns indicate the position of the 25th, 50th and 75th percentile in the ranked gene list which comprises 5224 genes and is based on the data set of van't Veer *et al*.. The last column indicates the number of genes in each category. The full table is available as a supplementary file [see Additional file 3].

GoName	GoId	WRS		K-S	min-*p*	25%	50%	75%	#genes
		s.l.p.	g.p.						
carbon-nitrogen ligase act..	0016884	1	7	53	16	6	6	27	2
spindle microtubule	0005876	2	13	54	19	38	38	42	2
organic acid metabolism	0006082	3	9	11	8	564	1619	3283	83
carboxylic acid metabolism	0019752	4	8	12	7	564	1619	3283	83
intramolecular isomerase act..	0016863	5	10	10	12	195	412	453	4
deoxynucleoside kinase act..	0019136	6	18	60	51	70	70	117	2
GMP synthase activity	0003921	7	27	317	78	6	6	6	1
intramolecular isomerase act..	0016860	9	12	9	14	195	453	905	9
biotin metabolism	0006768	10	21	62	58	76	76	132	2
nucleus	0005634	71	16	28	10	1100	2353	3797	574
mitotic cell cycle	0000278	121	1	3	1	346	1419	3031	93
M phase	0000279	107	2	1	2	251	848	1862	41
nuclear division	0000280	124	3	2	3	251	867	1862	40
M phase of mitotic cell cycle	0000087	130	4	4	4	238	848	1862	36
mitosis	0007067	152	5	5	5	235	848	1862	35
cell cycle	0007049	142	6	6	6	731	1689	3599	172
cell proliferation	0008283	112	11	7	9	891	1947	3645	264
regulation of cell cycle	0000074	153	29	8	15	731	1689	3604	105
