# Supplementary material for: Comparing functional annotation analyses with Catmap
Source: BMC Bioinformatics. 2004 Dec 9;5:193. doi: 10.1186/1471-2105-5-193 (PMC543458; doi:10.1186/1471-2105-5-193)
Supplement: Additional File 1 — p-values for the Wilcoxon rank sum score. [file 1471-2105-5-193-S1.pdf]

Additional file 1
for the paper
Comparing functional annotation analyses with Catmap

June 15, 2004

p-values for the Wilcoxon rank sum score.

We consider a ranked list of genes containing N unique genes and a category of n genes from this list. In the general case there could be repetitions of genes in the ranked list. However, one might rename the genes to make them unique, remembering at the same time to enlarge the category if necessary. The ranks in the ranked list go from 1 to N. When the category genes are mapped to the ranked list each of them obtain a rank:

$$Rank(i) \quad , \quad i = 1, \ldots, n$$

Rank sum and ROC area

We want a measure of how much the category genes are located towards the top of the ranked list. Here the top means genes with low ranks, i.e. close to 1. One measure is the Wilcoxon rank sum, R, defined as:

$$R = \sum_{i=1}^{n} Rank(i)$$

The rank sum is located in the interval from $\sum_{i=1}^{n} i = \frac{n(n+1)}{2}$ to $\sum_{i=1}^{n}(N - n + i) = \frac{n(2N-n+1)}{2}$. Another measure is the ROC area (Receiver Operating Characteristic) defined as follows: Take all pairs of genes (g_1, g_2), where g_1 is one of the n category genes and g_2 is one of the $N - n$ genes from the list but not from the category. There are $n(N - n)$ such pairs. Find all pairs

(g_1, g_2) where the rank of g_1 is lower than g_2. Call the number of such pairs num. The ROC area is then the fraction of all pairs with g_1 having lower rank than g_2.

$$ROC = \frac{num}{n(N-n)}$$

We see that ROC is a number between 0 and 1. Higher ROC means that the category genes are located more towards the top of the ranked list. There is a relation between the rank sum, R, and the ROC area, ROC, which comes about as follows: For category gene i with $Rank(i)$ there are $N - Rank(i)$ genes with higher rank. Some of these however could be category genes. The sum $\sum_{i=1}^{n}(N - Rank(i))$ counts the total number of correctly ordered pairs except that pairs of category genes are included as well. To get num we should subtract the number of pairs of category genes, of which there are $\frac{n(n-1)}{2}$. Hence

$$ROC = \frac{\sum_{i=1}^{n}(N - Rank(i)) - \frac{n(n-1)}{2}}{n(N-n)} = \frac{\frac{1}{2}n(2N-n+1) - R}{n(N-n)}$$

Since ROC area and rank sum are equivalent it makes no difference which one we work with. We have chosen to give the ROC area as the output of the program since it has the advantage that it is always normalized in the interval from 0 to 1. For the calculation of p-values, discussed below, it is easier to use the rank sum.

p-value

Now we turn to the calculation of p-values. For a given ranked list and category with a rank sum R the p-value is the probability that a random ranked list and the same category produces a smaller rank sum than R. The p-value depends on the distribution (null hypothesis) from which ranked lists are drawn. For most null hypotheses it would be necessary to draw a large set of ranked lists, calculate the rank sums and count the number of times it was smaller than R. However for the null hypothesis of randomly permuted genes it can be done analytically. Randomly permuting the ranked list and mapping the category genes to the list is the same as selecting the ranks of the category genes randomly. In other words the ranks, $Rank(i)$, are n distinct numbers in the interval from 1 to N, randomly drawn. The total number of such configurations is $\binom{N}{n}$. Let $A(N, n, R)$ be the number of such configurations where the rank sum is less than or equal to R. The p-value is then

$$p = \frac{A(N, n, R)}{\binom{N}{n}}$$

We use three different methods for calculating the p-value analytically.

Iterative method

The iterative method is exact. It employs the fact that

$$A(N, n, R) = A(N-1, n, R) + A(N-1, n-1, R-N)$$

which follows from the observation that either rank N is occupied by a category gene or not. If rank N is not occupied by a category gene, the n category genes are sitting in a list of length $N-1$ giving rise to the first term. If rank N is occupied by a category gene, the $n-1$ others must be located in a list of length $N-1$ with a rank sum less than or equal to $R-N$. This relation can be repeatedly used to reduce the parameters to trivial cases. The $A(N, n, R)$ found in this way is exact but the method is slow for large parameters.

Gaussian

For large parameter values the distribution of the rank sum is approximated by a Gaussian distribution (Troyanskaya *et al.*, 2002, Walpole *et al.*, 1998). Only the mean and variance of the rank sum needs to be known in order to use the Gaussian approximation. The mean is easily found:

$$E(\text{rank sum}) = E(\sum_{i=1}^{n} Rank(i)) = \sum_{i=1}^{n} E(Rank(i)) = \sum_{i=1}^{n} \frac{N+1}{2} = \frac{n(N+1)}{2}$$

The variance can be found by a similar but more tedious calculation to be

$$Var(\text{rank sum}) = \frac{n(N-n)(N+1)}{12}$$

The p-value is approximated by the probability to draw a smaller number than the real rank sum from a Gaussian distribution with this mean and variance. The Gaussian approximation is very fast but can deviate from the exact result. In our experience, it deviates most in the tail of the distribution, which is where the most significant p-values are found. However, it is usually not important whether the p-value is 10^{-10} or 10^{-12}. Qualitatively the Gaussian approximation gives the correct result.

Volume approximation

The volume approximation is used for cases where the exact method is too slow and the Gaussian result needs to be improved. It works as follows. Take a coordinate system with n

coordinates. Each coordinate corresponds to a category gene. Consider points in the coordinate system with integer coordinates, each of which is between 1 and N, and no two coordinates of the point are equal. Such allowed points are in exact correspondence with a set of ranks of a category. Draw a hyperplane in the coordinate system with the equation

$$\sum_{i=1}^{n} x_i = R$$

The number $A(N, n, R)$ is then given by the number of allowed points below the hyperplane. The volume approximation replaces the number of allowed points below the hyperplane with the volume below the hyperplane and the total number of allowed points with the volume of the n dimensional hypercube. This approximation introduces two errors. Firstly it ignores that no two ranks must be equal, secondly there is a boundary error at the hyperplane because of the integrality of the points. The first error is small if n is small compared to N. If n is close to N one can consider the complement of the category genes instead. The second error is small unless R is very close to its minimum.

After rescaling with a factor of N the approximate p-value is equal to the volume of the intersection of the n dimensional hypercube and the points below the hyperplane with the equation

$$\sum_{i=1}^{n} x_i = r$$

Here $r = \frac{R}{N}$ is a number between 0 and n. Call this volume $V_n(r)$. For small n it is readily calculated:

$$V_1(r) = \begin{cases} 0 & \text{if } r \leq 0 \\ r & \text{if } 0 \leq r \leq 1 \\ 1 & \text{if } r \geq 1 \end{cases}$$

and

$$V_2(r) = \begin{cases} 0 & \text{if } r \leq 0 \\ \frac{1}{2}r^2 & \text{if } 0 \leq r \leq 1 \\ -\frac{1}{2}r^2 + 2r - 1 & \text{if } 1 \leq r \leq 2 \\ 1 & \text{if } r \geq 2 \end{cases}$$

It is, of course, generally true that $V_n(r) = 0$ if $r \leq 0$ and $V_n(r) = 1$ if $r \geq n$. The volume can be written as the value of an integral

$$V_n(r) = \int_0^1 dx_1 \cdot \cdot \int_0^1 dx_n \; \Theta(r - (x_1 + ..x_n))$$

where $\Theta(x)$ is the indicator function,

$$\Theta(x) = \begin{cases} 1 & \text{if } x \geq 0 \\ 0 & \text{if } x < 0 \end{cases}$$

Using the fact that $\frac{d\Theta(x)}{dx} = \delta(x)$ it is easily derived that

$$\frac{dV_n(r)}{dr} = V_{n-1}(r) - V_{n-1}(r-1) \tag{1}$$

Using this formula and induction in n and the form of V_1 or V_2 it is seen that $V_n(r)$ is a piecewise polynomial function. The polynomials are pieced together at integer values of r. Let $V_{n,k}$ be the polynomial that gives V_n in the interval $[k; k+1]$ where k is an integer between 0 and $n-1$. Again using induction in n and the above equation it is furthermore seen that $V_{n,k}$ is a polynomial of degree n. We can thus write $V_{n,k}$ as

$$V_{n,k}(r) = \sum_{j=0}^{n} \frac{c_{n,k,j}}{n!}(r-k)^j$$

We have expanded the polynomial in $r-k$ instead of r and introduced $n!$ for convenience, as will be clear below. In order to calculate the volume it is necessary to know the coefficients $c_{n,k,j}$. One way to find them is to start with $n=1$ and repeatedly use eq.(1). However it can be done faster by noting that V_n is $n-1$ times continuously differentiable. This can again be proven by induction using eq.(1). $V_{n,k}$ can now almost be derived from $V_{n,k-1}$ using continuity of the function and the first $n-1$ derivatives in the point $r=k$ where these two polynomials glue together. Actually $c_{n,k,j}$ is the j derivative in $r=k$ which explains the form of the expansion of $V_{n,k}$. This determines the coefficients $c_{n,k,j}$ for $j = 0,..,n-1$. The n'th degree coefficient $c_{n,k,n}$ is not determined by continuity. However it can be derived exactly. By matching $(n-1)$'th degree coefficients in eq.(1) one gets

$$c_{n,k,n} = c_{n-1,k,n-1} - c_{n-1,k-1,n-1}$$

Using the result for $n=1$ and induction this equation has the unique solution

$$c_{n,k,n} = (-1)^k \binom{n-1}{k}$$

Here the famous relation from Pascal's triangle

$$\binom{n}{k} = \binom{n-1}{k} + \binom{n-1}{k-1}$$

was used. The polynomial $V_{n,0}$ is easily found either by matching to the region $r \leq 0$ or by induction or by direct calculation of the integral.

$$V_{n,0}(r) = \frac{1}{n!}\, r^n$$

The procedure to find the volume for parameters n, r is now the following. Let k be the integer part of r. Start with $V_{n,0}$ as above and use it to find $V_{n,1}$ by continuity of the function and the $n-1$ derivatives in the point $r=1$. Then find $V_{n,2}$ and so on until $V_{n,k}$ is found. The volume is then given by $V_{n,k}(r)$.

Choice of method to calculate the p-value

We need to choose between the 3 methods given the values of the 3 parameters: the length of the ranked list, N, the number of genes in the category n and the rank sum R. The choice is based on a balance between accuracy and speed. The iterative method is exact and is used whenever it is fast enough. By counting the number of variables, that needs to be calculated, the time complexity of the 3 methods can be estimated. For the iterative method the time is proportional to $N \cdot n \cdot R$. For the volume approximation it is proportional to $n^2 \cdot \frac{R}{N}$. The Gaussian method is for all practical purposes fast. The choice of method is as follows: If $N \cdot n \cdot R \leq 10^6$ the iterative method is used since it is exact. In the special case $R < N$ the rank sum effectively sets a cutoff on the number of relevant genes and the iterative method is still used provided $n \cdot R^2 \leq 10^6$. If not the Gaussian p-value, p_{gauss} is calculated. If $p_{\text{gauss}} > 0.1$ it is used. This is because the Gaussian is reliable away from the extreme tail of the distribution. If $p_{\text{gauss}} \leq 0.1$ and $n^2 \cdot \frac{R}{N} \leq 10^5$ the volume method is used. In the case that none of these conditions are satisfied the Gaussian result is given, even though it might be imprecise. The imprecision is not really serious however. Typically it could be the question of whether a p-value is 10^{-6} or 10^{-8}. For any reasonable parameters it would never make a very significant category insignificant or vice versa. This finishes our description of the score functions and the algorithm for finding p-values.

References

Walpole, R.E., Myers, R.H. and Myers, S.L. (1998) Probability and Statistics for Engineers and Scientists, sixth edition *Prentice Hall International, New Jersey.*

Troyanskaya, O. G., Garber, M. E., Brown, P. O., Botstein, D. & Altman, R. B. (2002) Nonparametric methods for identifying differentially expressed genes in microarray data. *Bioinformatics,* **18**, 1454-61.
